# Meta-Enrichment Analyses to Identify Advanced Gastric Cancer Patients Who Achieve a Higher Response to S-1/Cisplatin

**DOI:** 10.3390/cancers11060871

**Published:** 2019-06-21

**Authors:** Madoka Takeuchi, Jaffer A. Ajani, Xuemin Fang, Per Pfeiffer, Masahiro Takeuchi, Hanneke W. M. van Laarhoven

**Affiliations:** 1 Faculty of Environment and Information Studies, Keio University, 5322 Endo, Fujisawa-shi, Kanagawa 252-0882, Japan; 2 Gastrointestinal Medical Oncology, MD Anderson Cancer Center, Houston, TX 77030, USA; jajani@mdanderson.org; 3 Clinical Medicine (Biostatistics), Kitasato University, Tokyo 108-8641, Japan; mindy.fang@gmail.com (X.F.); tsmasaamg@gmail.com (M.T.); 4 Experimental Research in Medical Cancer Therapy, Odense University Hospital, 5000 Odense C, Denmark; per.pfeiffer@rsyd.dk; 5 Department of Medical Oncology, Cancer Center Amsterdam, Amsterdam University Medical Centers, University of Amsterdam, 1105 AZ Amsterdam, The Netherlands; h.vanlaarhoven@amc.uva.nl

**Keywords:** predictive enrichment strategy analysis, FLAGS trial, DIGEST trial, gastric cancer, S-1

## Abstract

The Multicenter phase III comparison of cisplatin/S-1 with cisplatin/infusional fluorouracil in advanced gastric or gastroesophageal adenocarcinoma study (FLAGS) and the Diffuse Gastric and Esophagogastric Junction Cancer S-1 Trial (DIGEST) have shown that patients with advanced gastric cancer treated with S-1/Cisplatin (CS) have similar overall survival (OS) compared to 5-fluorouracil/cisplatin (CF). The purpose of this analysis was to identify patients who may specifically benefit from CS using meta-enrichment analysis of the combined two datasets. Eleven clinico-pathological factors were selected and a high response enrichable population was determined. The efficacy of CS in the combined data set of 1365 patients (*n* = 1019 from FLAGS and *n* = 346 from DIGEST) was analyzed. We identified 683 patients (*n* = 374 from CS, *n* = 309 from CF) as the high response enrichable population who were classified as those with Eastern Cooperative Oncology Group Performance Status (ECOG PS) 1, more than two metastatic sites and low neutrophil-lymphocyte ratio (log(NL ratio)). In the high response enrichable population, the median OS in the CS group was 241 days compared to 210 days in the CF group (hazard ratio 0.776; 95% confidence interval 0.658 to 0.915; *p*-value 0.004). Through meta-enrichment analysis, the high response enrichable population to CS was identified. Our findings show the clinical importance of selecting the appropriate treatment based on specific patient characteristics.

## 1. Introduction

Gastric cancer is the fifth most common cancer in the world, and it has been estimated that over 700,000 people will succumb to it every year [1]. While there have been improvements in diagnosis and treatment, outcomes remain poor compared to those of other cancer types with similar incidence, such as breast cancer and colon cancer [2]. To date, a variety of clinical trials have been performed in different countries incorporating different first-line treatment regimens. Generally speaking, doublet regimens are used in Asian countries and while triplet regimens are also in use in Europe and the United States, a first-line chemotherapy approach based on 5-FUs and platinums has been established as the standard treatment worldwide for unresectable or recurrent gastric cancers [3,4,5].

Given the marginally improved survival benefit of triplets compared to doublets, as well as the significantly increased toxicity, doublets are generally considered to be the standard of care for the general population of advanced esophagogastric cancer patients [6,7]. However, the question of whether a specific doublet should be preferred for a specific group of patients in terms of survival and/or adverse effects is still unanswered.

Two phase III trials were conducted to evaluate the benefit of the doublet therapy of cisplatin/ S-1 compared to cisplatin/infusional fluorouracil. The FLAGS trial, a multi-center, phase 3 study conducted in Europe and the United States, evaluated the superiority of cisplatin + S-1 (CS) to cisplatin + 5-FU (CF) in overall survival (OS) for the treatment of advanced gastric and gastroesophageal junction cancer. In the FLAGS trial, there was no significant difference in OS, however the safety and toxicity profiles were more favorable for CS. Data from the FLAGS study led to the approval of CS for treatment of advanced esophagogastric cancer by the European Medicines Agency (EMA) [8,9]. A subsequent meta-analysis of data available thus far, showed that S-1 is effective and tolerable as first-line therapy for advanced gastric cancer in both Asian and Western countries [10]. Additional analysis of the FLAGS data showed that CS was non-inferior to CF in OS, and that CS may be more effective and safer for patients with diffuse type gastric cancers [11].

The DIGEST trial evaluated the superiority of CS to CF in OS for patients with advanced diffuse type gastric and gastroesophageal junction cancer. CS was not superior to CF in OS and both therapies had similar efficacy and safety [12]. Thus, CS and CF are both valid treatment options for clinical use in Europe.

Given the suggestion from the FLAGS data regarding that a specific subgroup of patients could particularly benefit from CS [11], we aimed to combine data sets from both the FLAGS and the DIGEST trials to identify a population in which CS was more effective than CF. For this purpose, we used predictive enrichment strategy analysis (PESA), which is a novel statistical method for identifying a treatment effective population based on clinical trial results. PESA, a new concept with guidelines by the US Food and Drug Administration (FDA), is a systematic, pre-specified procedure to identify the patient population that will benefit most from a particular treatment. [13,14]. Enrichment analysis is “the prospective use of any patient characteristic to select a study population in which detection of a drug effect (if one is in fact present) is more likely than it would be in an unselected population” [14].

## 2. Results

### 2.1. Creating Individual Enrichment Scores

The efficacy in the combined dataset of 1365 patients (*n* = 1019 from FLAGS and *n* = 346 from DIGEST) was analyzed to create individual enrichment scores. After discussion with additional opinion leaders, we removed 5 clinico-pathological factors (heart rate, respiratory rate, lymphocyte, hemoglobin and temperature). Eleven clinico-pathological factors (ECOG performance status, disease type, liver metastasis, lung metastasis, diffuse type, age, gender, albumin, primary region, neutrophil lymphocyte (N/L) ratio and trial) were reselected and a high response enrichable population was determined (Table 1). Due to the skewed nature of the N/L ratio, the variable was transformed to the log scale to become a normal distribution.

Subjects with missing values for the selected factors were removed from the analysis; 69 subjects had missing Albumin values and 24 subjects had missing N/L ratios.

### 2.2. Selecting the Enrichment Subgroup

The Cox two lasso true scoring system was chosen as the most optimal system. The maximum score was 0.15, resulting in a z-test score value of 2.2. However, with a score of 0.15, the population would be divided in an 85:15 ratio, creating an imbalance. Since a score of 0.5 had a similar z-score test value, 0.5 was chosen as the threshold value. The score of 0.5 meant that the enrichable population would consist of subjects whose scores were approximately among the top 50% of the total analyzed population.

### 2.3. Validation Enrichment Subgroup and Patient Mapping

**Figure 1:**
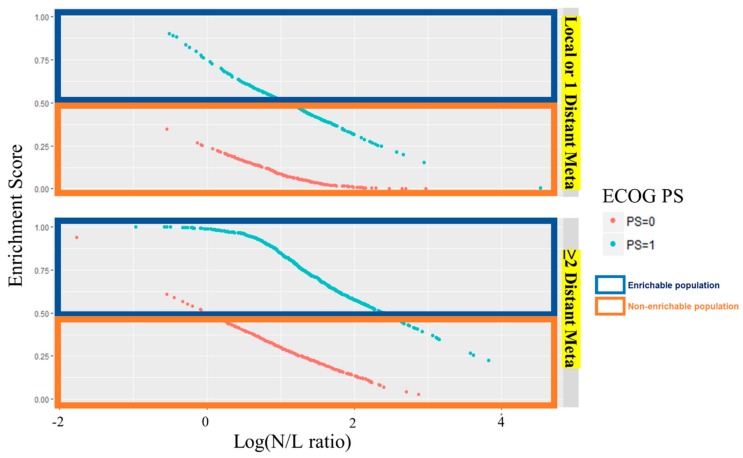
ECOG PS, eastern cooperative oncology group performance status; N/L, neutrophil/lymphocyte; Meta, metastasis.

A total of 683 patients, 374 from FLAGS and 309 from DIGEST, were identified as the high response enrichable population. After patient mapping, high response patients were classified as patients with ECOG PS 1, more than two metastatic sites and a low neutrophil-lymphocyte ratio (log(N/L ratio)) (Figure 1).

**Figure 2:**
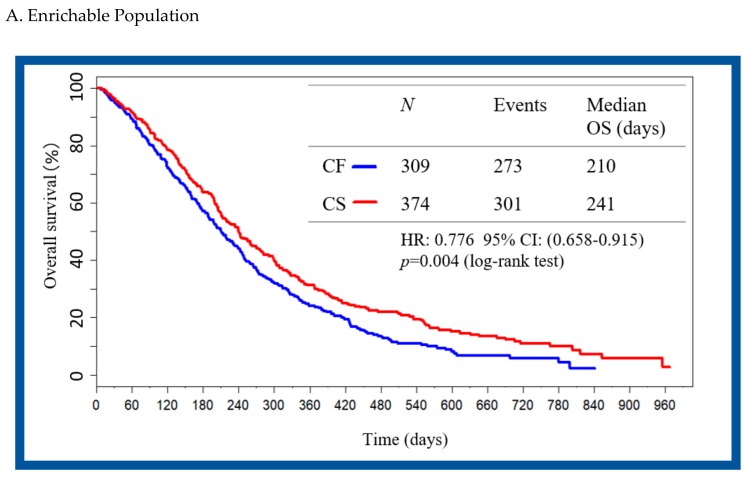

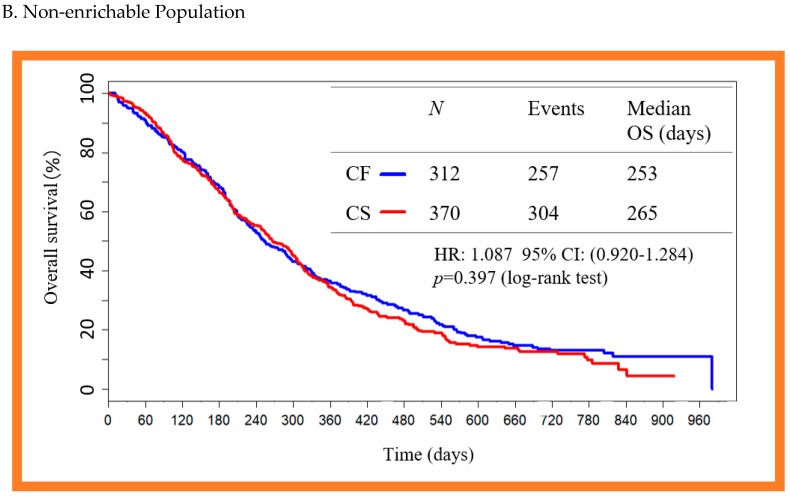
Kaplan–Meier plot of overall survival for the enrichable and non-enrichable population. (**A**) Enrichable population. (**B**) Non-enrichable population. CF, 5-fluorouracil/cisplatin; CS, S-1/Cisplatin; CI, confidence interval; HR, hazard ratio; OS, overall survival.

In the high response enrichable population, the median overall survival in the CS group was 241 days compared to 210 days in the CF group (HR 0.776; 95% CI 0.658 to 0.915; *p*-value 0.004). In the non-enrichable population, the median overall survival in the CS group was 265 days and 252 days in the CF group (HR 1.087; 95% CI 0.920 to 1.284; *p*-value 0.397) (Figure 2).

## 3. Discussion

This research study is the first to report findings based on predictive enrichment strategy analysis using two prospective, global, phase 3 clinical trials. Subjects who had “PS = 1”, “2 or more metastases” and “low neutrophil to lymphocyte ratio” were identified as the “enrichable population”. For this enrichable population, CS prolonged survival more than CF. This finding shows the clinical importance of selecting the appropriate treatment based on patient characteristics.

Our results identified “PS = 1” as a factor contributing to better OS of CS [8,12]. In both the FLAGS and DIGEST trials, patients could be included if they had a performance status of 0 or 1. In previous publications, those treated with CS had less severe adverse events and fewer treatment discontinuations due to adverse events compared to CF [11], thus CS may be appropriate for symptomatic patients, characterized as “PS = 1”. Importantly, patients with a high number of metastases generally have a poor prognosis [15]. In our analysis, in addition to PS = 1, we also identified “2 or more metastases” as an enrichable factor.

Although based on PS and the number of metastases, one could argue that CS may be most appropriate for a relatively poor prognosis patient population, this idea counters our finding that in patients with low neutrophil to lymphocyte ratio (NLR), CS was more effective. NLR has been reported to be useful as an indicator of immune function and gastric cancer patients with lower NLRs have a more favorable prognosis [16,17].

Furthermore, the survival curves of CS and CF in the non-enrichable population (Figure 2) show similar trends and no significant difference in outcomes. This result may be clinically meaningful, since CS was shown to have fewer adverse events compared to CF. Thus, in the non-enrichable population, CS may be a more favorable treatment option than CF.

The combination of poor and good prognostic factors that are required to select a specific patient population who may benefit the most from CS, underscore the relevance of the PESA approach and the need for clinically applicable prediction models to guide treatment decisions [18].

Several limitations of this study should be acknowledged. First, this meta-enrichment analysis is a post hoc, secondary analysis of existing data to identify a potential population likely to benefit from CS, and thus the analysis was not stated prior to the preliminary data examination. Furthermore, meta-enrichment analysis is a novel analysis technique for advanced gastric cancer as well as other diseases, and there are limited reports using enrichment analysis to identify a patient population likely to benefit from therapy. Thus, it is difficult to compare this analysis with previously reported analyses. Second, in the enrichment analysis, selection of the clinico-pathological factors was subjective with no clear guidelines on how to select these factors and, therefore, the “enrichable” population may differ based on the selected factors. Although peritoneal metastasis is a clinically meaningful factor, this variable was not used in the analysis due to the limited quality of the data collected. Finally, to perform the “meta” enrichment analysis, the FLAGS and DIGEST trial data were combined. Statistically, the trial variable was not significant, however, the patient population in DIGEST was limited to diffuse-type patients while the FLAGS trial included both diffuse (*n* = 590) and non-diffuse type (*n* = 439) patients, reflecting the different patient populations in the two trials.

Some strengths should also be mentioned. As in any disease area, treatment effects are not homogenous across the gastric cancer population. Subgroup analysis of the patient population may identify a patient characteristic more likely to benefit from the treatment, however, multiple subgroup analyses often result in inflation of type I error and lower power. Enrichment analysis allows for several clinico-pathological factors to be analyzed simultaneously, decreasing the potential statistical issues that arise from subgroup analysis.

## 4. Materials and Methods

Innovative statistical methods and analysis have been developed to further identify the patient sub-population most likely or expected to benefit from the treatment. Harvard University’s working paper’s three step PESA was applied to the FLAGS and DIGEST data sets to identify an enrichable population likely to benefit from CS [13].

In the initial analysis, discussion based significant clinico-pathological factors were selected. The selected factors were; ECOG PS, disease type, liver metastasis, peritoneal metastasis, tissue type, heart rate, respiratory rate, neutrophil count, albumin, lymphocyte count, primary region, hemoglobin levels, gender and body temperature (Table 2). These 14 factors were used to create scoring systems in the FLAGS dataset and the scoring systems were then compared in the DIGEST dataset. The FLAGS dataset was used as the training set and the DIGEST trial dataset was used as the validation set.

It was later found that the evaluation method for the presence of peritoneal metastasis was different in the two trials. The FLAGS trial had both individual and central review committee assessments, and there were large discrepancies between the two assessments (Table 3). The DIGEST trial only consisted of individual assessment of the metastasis. Although peritoneal metastasis is an important clinico-pathological factor, due to possible data inconsistencies, we decided to remove the presence of peritoneal metastasis from the analysis. In addition, several factors that may not have been clinically relevant, such as heart rate and respiratory rate, were removed.

In addition, to overcome differences in baseline characteristics of the FLAGS and the DIGEST, meta-enrichment analysis was performed by combining the data from the two trials. To take the treatment effect of the two trials into account, a “trial” variable was added to the analysis. The “trial” variable was not significant, which resulted in consistent treatment effects in the FLAGS and DIGEST trials which further validated the combination of the two datasets. The combined datasets thus allowed for meta-enrichment analysis to identify a robust subgroup of high response patients.

Taking into account the differing assessment methods and reselection of clinico-pathological factors, an updated PESA was performed.

### 4.1. Creating Individual Enrichment Scores

After combining the data, individual enrichment scores were created based on selected clinico-pathological factors. To select the clinico-pathological factors, all possible meaningful factors related to advanced gastric cancer were listed and then given to specialists and opinion leaders to identify the most clinically important factors. After selection of factors, half of the data were randomly selected as the training set and the other half as the validation set. Using the training set, six scoring systems using the prediction models based on various Cox proportional hazards model with the selected clinico-pathological factors were created. Each subject was to have a score representing an estimated treatment difference. The higher the score of the patient, the more likely the patient would benefit from CS. Each individual score reflected an anticipated average treatment difference for future patients who may have a similar baseline profile. Higher scores indicated that the patients tended to benefit from the new therapy.

### 4.2. Selecting the Enrichment Subgroup

The scoring systems were then compared using the validation set. The most optimal Cox proportional hazards model was selected, and the maximal score was determined. This procedure was cross validated 2000 times.

### 4.3. Validation Enrichment Subgroup and Patient Mapping

After the scoring system and maximal score were determined, patient mapping of the baseline characteristics was done to look at the characteristics that best described the enrichable population.

### 4.4. Statistical Analysis

Survival rates for the enrichable and non-enrichable population were summarized using Kaplan-Meier curves and were further characterized in terms of median survival time (MST) and 95% confidence interval (CI). The *p*-value of the two sample log-rank test was given.

## 5. Conclusions

In this analysis we were able to identify a population that had superior survival with CS compared to CF. This study may be helpful in clinical practice with selection of the most appropriate regimens based on a patient’s background and characteristics.

## Figures and Tables

**Table 1:** Re-selection of the clinico-pathological factors in FLAGS and DIGEST trial.

Covariate	Number of NA	Data Details
PS	0	PS 0 or PS 1
Disease Type	0	(Local or 1 Distant Meta) or (≧2 Distant Meta)
Baseline site	0	Liver meta (+/−)
	0	Lung meta (+/−)
Tissue Type	0	Diffuse Type or Not Diffuse Type
Age	0	Continuous
Gender	0	Male or Female
Albumin	69	Continuous
Primary Region	0	Stomach (+/−)
N/L ratio	24	Continuous
Trial Label	0	Indicator of the FLAGS or DIGEST

NA, not available; N/L, neutrophil/lymphocyte; Meta, metastasis; PS, performance status.

**Table 2:** Selection of the 14 clinico-pathological factors in FLAGS trial.

Covariate	Number of NA	Data Details
PS	0	PS 0 or PS 1
Disease Type	1	(Local or 1 Distant Meta) or (≧2 Distant Meta)
Baseline site	0	Liver meta (+/−)
	0	Peritoneal meta (+/−)
Tissue Type	0	Diffuse Type or Not Diffuse Type
Heart Rate	24	Continuous
Respiratory Rate	95	Continuous
Neutrophil	1	Continuous
Albumin	44	Continuous
Lymphocyte	9	Continuous
Primary Region	0	Stomach (+/−)
Hemoglobin	1	Continuous
Gender	0	Male or Female
Temperature	34	Continuous

NA, not available; Meta, metastasis; PS, performance status.

**Table 3:** Discrepancy between central and individual investigator assessment in the FLAGS trial.

**FLAGS Trial**		**Investigator Assessment (Peritoneal Metastasis)**	
		No	Yes
**Central assessment** **(peritoneal metastasis)**	No	495	26
	Yes	402	106
